# Infertility’s Hidden and Evident Dimensions: A Concern Requiring Special Attention in Iranian Society

**Published:** 2019-11

**Authors:** Zahra KIANI, Masuomeh SIMBAR

**Affiliations:** Midwifery and Reproductive Health Research Center, Shahid Beheshti University of Medical Sciences, Tehran, Iran

## Dear Editor-in-Chief

Infertility, defined as a failure to achieve pregnancy after one year of sexual intercourse without contraception, is a global problem (1). Its prevalence increased by around 50% compared with the levels observed in the previous decade. Globally, 50% of infertility cases are accounted for by male-related factors, while 25% to 35% are due to blocked fallopian tubes in women (2). For several years, infertility was identified by WHO as one of the most pressing global health problems. The organization collects infertility data and releases international laboratory and diagnostic standards for infertility. Unfortunately, however, the condition has not been regarded as seriously by other organizations like WHO. Some of the most important philanthropic and nongovernmental institutions, along with international organizations devoted to fertility health, do not support infertility care and do not consider it a significant issue in developing countries (3).

In the context of Iran, 10.6% of Iranian couples experience infertility throughout their lives, thus rendering the condition a key challenge for husbands and wives. The causes of infertility in the country include male-related factors (34.0%), female-associated determinants (43.5%), a combination of the first two factors (17.1%), and unknown reasons (8.1%). The global infertility rate is lower than the prevalence of primary infertility in Iran, where the absence of ovulation is the major cause of the condition (4). In developing countries, many religions highly emphasize fertility and birth as children are very valuable because of social, cultural, and economic reasons (5).

Infertility adversely influences many aspects of life, such as sexual relationships, quality of life, and mental health. It causes depression, anger, anxiety, and fear, which can interfere with the manner by which people live. People afflicted with infertility are also confronted with stressful problems, including costly treatments, repeat visits to physician, tests and examinations, time wastage, the provision of a full history to physicians, and the determination of a specific plan for intercourse (6, 7).

The failure to have a child can impose considerable pressure on the relationships of couples and diminish the appeal of sexuality. Because of illogical thoughts, people end their sexual relationships with their spouses and attempt to reduce their sexual desires. Sexual desire is a primary life issue that can affect the personal and social lives of women and their spouses. Sexual problems can cause psychological imbalance, resulting in separation and, eventually, divorce (8). Throughout society, many infertile women and men likewise suffer from social isolation and despair. Their functioning is affected by society, families, and cultures, and they consider their social security to be at risk because the absence of offspring means that no one will look after them in old age or at times of disease. Infertility often eventually leads to economic difficulties given that couples without children usually spend their incomes on infertility treatments. Concerns about the growth of infertility and, in turn, the deficit in healthcare resources and infrastructures, the heavy burden of other life-threatening diseases, such as HIV/AIDS, and maternal mortality threaten societies (3). In addition, inexpensive diagnostic tests and treatments are rarely offered in public health systems; assisted reproductive technology (ART) is offered mostly in private clinics at high costs and is thus unavailable to many individuals (2).

Considering the severity of the above-mentioned problems, infertility is a situation that affects the basic human rights of individuals. Fertility health and infertility-related problems should, therefore, be included as components of human rights (9). In this regard, the International Conference in Bangkok (1988) and the International Conference on Population and Development (1994) emphasized infertility as a global problem and a source of serious damage to reproductive health (10). In line with this perspective, as well, WHO recommended the integration of infertility care into primary health services (2).

In recently formulated population policies in Iran, two important solutions for increasing the country’s population are paying attention to couples who are capable of fertility, and the other side pay attention to infertile couples. Infertility is beyond a medical problem that interacts in a complex manner with social, economic, and cultural issues. The condition should be understood and managed, and its hidden and evident dimensions should be accorded special focus. Accordingly, we recommend that care facilities be provided to assist infertile individuals and enhance awareness about the consequences of infertility in society.
